# A Data-Driven Assessment of the Metabolic Syndrome Criteria for Adult Health Management in Taiwan

**DOI:** 10.3390/ijerph16010092

**Published:** 2018-12-31

**Authors:** Ming-Shu Chen, Shih-Hsin Chen

**Affiliations:** 1 Department of Healthcare Administration, Oriental Institute of Technology, New Taipei City 22061, Taiwan; tree1013@gmail.com; 2 Department of Information Management, Cheng Shiu University, Kaohsiung City 83347, Taiwan

**Keywords:** metabolic syndrome (MetS), decision tree, risk factors assessment, health management

## Abstract

According to the modified Adult Treatment Panel III, five indices are used to define metabolic syndrome (MetS): waist circumference (WC), high blood pressure, fasting glucose, triglycerides (TG), and high-density lipoprotein cholesterol. Our work evaluates the importance of these indices. In addition, we attempted to identify whether trends and patterns existed among young, middle-aged, and older people. Following the analysis, a decision tree algorithm was used to analyze the importance of the five criteria for MetS because the algorithm in question selects the attribute with the highest information gain as the split node. The most important indices are located on the top of the tree, indicating that these indices can effectively distinguish data in a binary tree and the importance of this criterion. That is, the decision tree algorithm specifies the priority of the influence factors. The decision tree algorithm examined four of the five indices because one was excluded. Moreover, the tree structures differed among the three age groups. For example, the first key index for middle-aged and older people was TG whereas for younger people it was WC. Furthermore, the order of the second to fourth indices differed among the groups. Because the key index was identified for each age group, researchers and practitioners could provide different health care strategies for individuals based on age. High-risk middle-aged and healthy older people maintained low values of TG, which might be the most crucial index. When a person can avoid the first and second indices provided by the decision tree, they are at lower risk of MetS. Therefore, this paper provides a data-driven guideline for MetS prevention.

## 1. Background

With economic growth and changes in diet and lifestyle habits, people are increasingly being diagnosed with metabolic syndrome (MetS). MetS is associated with gender, age, and obesity [1]. In the United States, 34% of adults met the criteria for MetS [2]. Moreover, men and women older than 60 years are more likely to have MetS. In São Paulo, Brazil, the prevalence of MetS in adults was 30.3% [3]. In Taiwan, the prevalence of MetS in women was markedly higher in postmenopausal women, for whom the prevalence exceeded that among men [4]. Some research evidences exist in Korea [5], Thailand [6], and Malaysia [7]. Thus, MetS is a global health risk, and thus places a substantial burden on health care [8]. Because MetS is highly correlated with other diseases [4,9,10,11,12], MetS management is key for adult health management.

People with MetS have increased risks of cardiovascular disease [10,13], type 2 diabetes mellitus [9], and stroke [11,12]. Hwang et al. demonstrated that MetS was highly correlated with overweight status and obesity [4]. In [3], the researchers used logistic regression models and reported that the glycemic index (GI) and dietary glycemic load were positively associated with high-density lipoprotein cholesterol (HDL-C) reduction in adult residents of São Paulo, Brazil.

Mohammadifard et al. [14] revealed that a high GI was significantly associated with MetS. Chen et al. [15] discovered that fasting glucose (FG), blood pressure (BP), triglycerides (TG), glutamate pyruvate transaminase, and white blood cell (WBC) count were the most critical risk factors. Ushida et al. [16] discovered that a combination of elevated γ-glutamyltranspeptidase and WBC count was the most significant combination of risk factors. Worachartcheewan et al. [6] demonstrated that the combination of TG, BP, and fasting plasma glucose (FPG) were strong predictors of MetS. Mohammadifard et al. [14] indicated that a high GI and FG were significantly associated with MetS. In summary, significant factors differ when different predictors are considered.

Upon careful review of the literature, we discovered some gaps in this area of study. We like to fill some gaps of existing research. First, we incorporated the five indices for MetS diagnosis defined by the modified Adult Treatment Panel III (ATP III) to evaluate across multiple age groups. The present study analyzes the five indices defined by the ATP III, identify the significant risk factors, and achieve early prevention of associated health complications for some age groups. Achievement of the aforementioned goals constitutes a significant contribution of this research.

Finally, because MetS is an increasingly significant health problem in Taiwan [17], we analyzed actual patient cases in Taiwan. The results could serve as a reference for other countries. The remainder of this paper is organized as follows. Because this study utilized the definition of MetS from the Ministry of Health and Welfare in Taiwan and a decision tree algorithm, we present the criteria and then introduce the decision tree algorithm in Section 2. In Section 3, we illustrate descriptive statistics related to MetS and show the results of the decision tree algorithm which extracts significant risk factors. Finally, this paper discusses the extracted risk factors and offers conclusions in Section 4 and Section 5, respectively.

## 2. Research Methods

### 2.1. Criteria of Metabolic Syndrome

According to the Health Promotion Administration, Ministry of Health and Welfare in Taiwan, the prevalence of MetS is estimated using the definitions of the modified ATP III and MetS criteria for Taiwanese people. The five major criteria shown in Table 1, can be used to determine whether a person has MetS. The first criterion is waist circumference (WC) according gender. If a man or woman has a WC of more than 90 or 80 cm, respectively, that person has an abnormal WC. The second criterion—high BP—includes the rates of systolic BP (SBP) and diastolic BP (DBP). If either the SBP or DBP is abnormal, the individual in question fits the criterion. The third criterion evaluates FPG. If the first FG test fails, secondary blood sugar data can be used. We selected secondary blood sugar data for this study. The fourth criterion is TG, where normal is defined as less than 150 mg/dL. If the TG value is larger than or equal to 150 mg/dL, the individual in question meets this MetS criterion. Finally, the fifth criterion is high density lipoprotein cholesterol (HDL-C). The modified ATP III states that if there are three or more of the following conditions: abdominal obesity, high TG, low HDL-C, hypertension, and hyperglycemia, this person meets the criteria for MetS. Table 1 describes the criteria in detail.

### 2.2. Data Source

This study analyzed datasets from the MJ Group—a major health screening center in Taiwan—from 2010 to 2015. The MJ Health-Check-Up-Based Population Database (MJPD) collected from four MJ clinics provides periodic health examinations to the center’s approximately 71,000 members and contains 201,087 cases. All of the datasets used in this study have been authorized by and received from the MJ Health Research Foundation (Authorization Code: AP_A2016002; Approval No.: MJHRF-2016005A). The data application procedures are described at http://www.mjhrf.org/main/page/release1/en/#release01. The MJPD database is accessible to academic researchers upon request. With respect to ethical issues regarding usage of data in the database, the protocol of this study was evaluated and deemed acceptable by the Research Ethics Review Committee at the Far Eastern Memorial Hospital (FEMH-IRB-107126-E) and the MJ Health Research Foundation.

### 2.3. Decision Tree Algorithm

The main differences among decision tree algorithms are the pruning strategy and the rule for splitting nodes [18]. A decision tree algorithm builds decision trees in a top-down recursive partitioning manner; that is, the attribute with the highest information gain (Equations (1)–(4)) is selected as the split node. This algorithm is run recursively until one class demonstrates that a clear majority does not generate a subproblem. Many decision tree algorithms apply the binary split.

In Equation (1), a subset of dataset D is the input, and its entropy is calculated as follows:

(1) $$\mathrm{Entropy}\left(D\right)=\sum_{i=1}^{c}P_{i}\times \log_{2}P_{i}$$

where *P_i_* is the probability of class *i* in the subset of data *D* and *C* is the number of classes. In Equation (2), *Gain* is the reduction in entropy when the value of attribute *A* in the dataset is known.

(2) $$\mathrm{Gain}\left(D,A\right)=\mathrm{Entropy}\left(D\right)-\sum_{j=1}^{v}\frac{\left|D_{j}\right|}{\left|D\right|}\mathrm{Entropy}\left(D_{j}\right)$$

where *v* is the number of classes of attribute *A* and |*D_j_*| is the number of observations with class *j* in the subset of data *D*. Equation (3) calculates the attribute of the normalized information gain.

(3) $$\mathrm{SplitInfo}\left(D,A\right)=\sum_{j=1}^{v}\frac{\left|D_{j}\right|}{\left|D\right|}\log_{2}\left(\frac{\left|D_{j}\right|}{\left|D\right|}\right)$$

Finally, Equation (4) evaluates the gain ratio of attribute *A*. A decision algorithm selects the attribute that exhibits the highest rate.

(4) $$GainRatio\left(A\right)=\frac{Gain\left(D,A\right)}{SplitInfo\left(D,A\right)}$$

## 3. Experimental Results

We purchased a dataset from a major health screening company in Taiwan called MJ Group. The dataset period was 2010–2015. This study was approved by the Research Ethics Review Committee and Institutional Review Board of the Far Eastern Memorial Hospital (IRB No. FEMH-IRB-106058-F, date: 20170501). The personal information of all subjects was removed to protect privacy. A total of 201,087 records with 77 variables were obtained, including the five MetS criteria and other details such as age, height, and occupation. Each subject was aged ≥20 years. We analyzed the data by using descriptive statistics, as described in Section 3.1. Section 3.2 describes the application of the decision tree to analyze the importance of the five MetS criteria for the whole dataset. We divided the dataset into three groups, namely, younger (age less than 45 years), middle-aged (age is greater than 45 and less than 65 years), and older (age is greater than or equal to 65 years) groups.

### 3.1. Descriptive Statistics

As listed in Table 2, the dataset contained 201,087 cases, namely, 87,289 younger cases, 100,170 middle-aged cases, and 13,628 older cases. The middle-aged group had the largest proportion of cases and the most patients with MetS. The younger and older groups had the lowest and highest MetS rates, respectively. In our dataset, 7% of the patients had MetS. A dataset in a prior study [2] showed that 34% of adults in the United States met the criteria for MetS. Thus, the MetS rate in Taiwan is lower than that in the United States.

**Figure 1:**
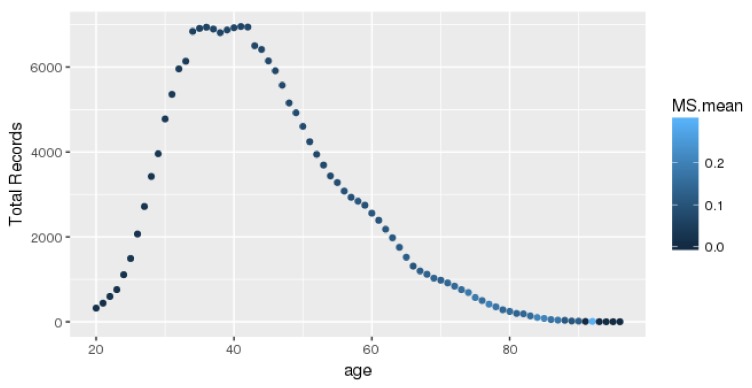
The MetS records of each age.

The records for each age group are presented in Figure 1. Each dot represents an age record with a different color. The number of complete records is over 6000 for the middle-age group. Our data were obtained from a health check-up company, and most of the subjects aged 35–40 years underwent annual examinations paid for by their companies. Clearly, the number of subjects over the age of 42 years was lower. In addition, each color represents the mean MetS rate of a different age. If the color is lighter, the average value of the age span in question is higher.

**Figure 2:**
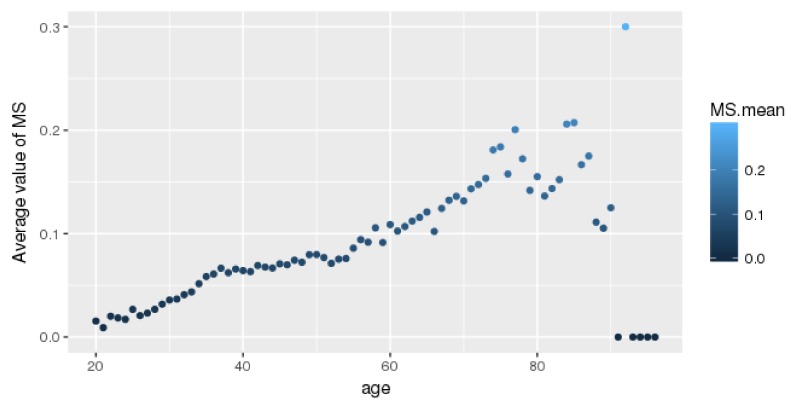
The average prevalence rate of metabolic syndrome (MetS) for all ages.

The average MetS value for each age is shown in Figure 2. Older age is associated with a higher likelihood of having MetS. Thus, elderly people should be aware of MetS. An increasing pattern similar to that demonstrated by Hwang et al. [2] is shown in Figure 2. Some exceptions over the age of 90 years were noted.

### 3.2. Decision-Making Model for the Whole Population

The five MetS criteria determine whether a person has MetS. These criteria are WC (g_wc), high BP (g_ssr), FPG (dm_fg), TG (l_tg), and HDL-C (l_hdlc). The importance of these factors is unknown when screening people with MetS. Determining the importance of these five factors could facilitate the development of guidelines for preventing this disease. This subsection describes the application of the decision tree technique, which filters characteristics based on information gain. The factor that provides the most distinguishable information is shown at the top of the tree. We ran the decision tree in R language.

**Figure 3:**
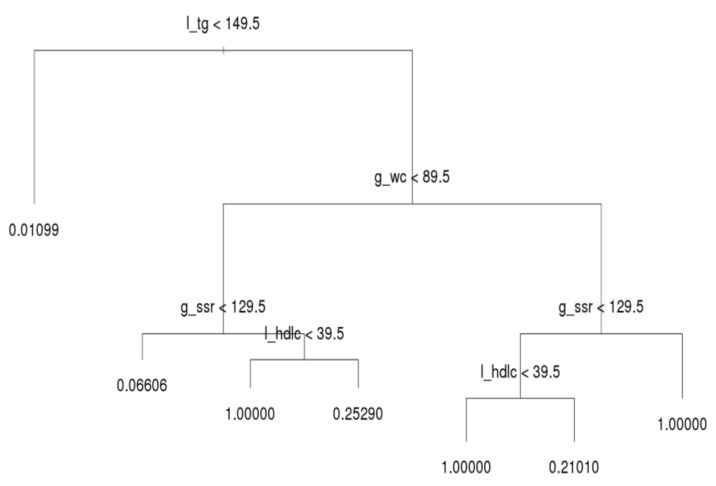
Decision tree for all ages.

After running the decision tree algorithm, we show the results of the whole dataset in Figure 3; l_tg, g_wc, g_ssr, and l_hdlc were included in the decision tree, whereas dm_fg was excluded. The implication is that l_tg, g_wc, g_ssr, and l_hdlc are major critical factors based on filtering of people with MetS. Most notably, l_tg is shown on top of the decision tree, and thus this criterion was deemed the most crucial factor for distinguishing MetS symptoms. If a person has a l_tg score lower than 149.5, he or she meets only 1.1% of the criteria for MetS. Hence, to prevent MetS, l_tg should be controlled.

The second and third most crucial factors for distinguishing MetS symptoms were g_wc and g_ssr, respectively. A person with high l_tg but low g_wc (lower than 89.5) has a 6.6% likelihood of having MetS symptoms. In the same subtree, a person with high g_ssr (higher than 129.5) and l_hdlc (higher than 39.5) has a 25.3% likelihood of having MetS symptoms. However, if such a person has low l_hdlc, he or she has MetS. Moreover, if g_wc is abnormal (e.g., higher than 89.5) and l_hdlc is normal, the individual in question has a 21% likelihood of having MetS. When g_ssr and l_hdlc are abnormal, the individual in question has MetS. In general, l_tg is the most crucial index for preventing MetS in all three age groups; g_wc, g_ssr, and l_hdlc are the second, third, and fourth most crucial criteria for determining MetS, respectively; dm_fg is the crucial factor that least influences the decision tree results. This subsection reveals the deciding factors for the whole population. Section 3.3 explains the differences among the three age groups.

### 3.3. Close Look at Age Groups

**Figure 4:**
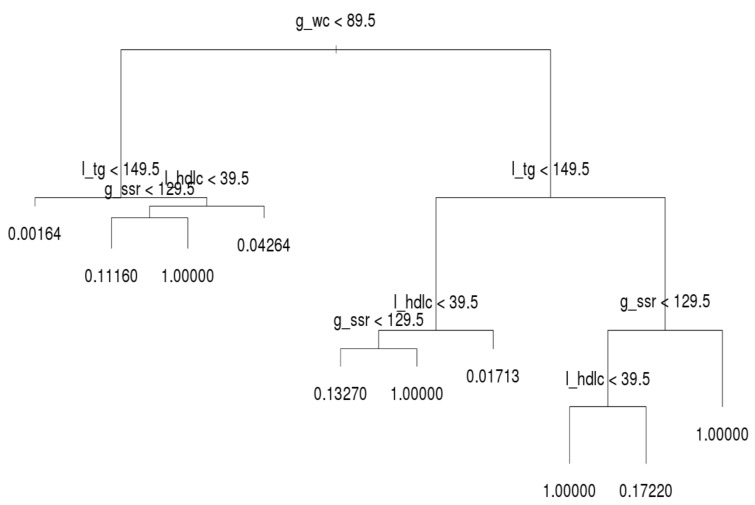
Decision tree for the younger group.

**Figure 5:**
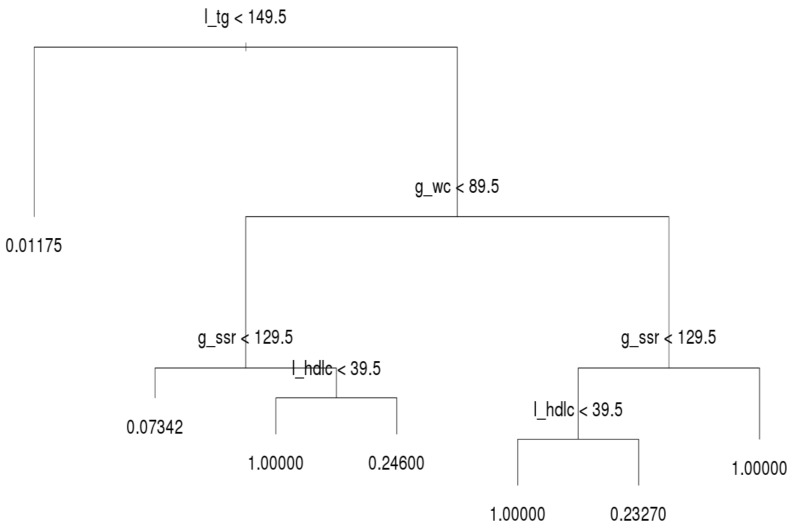
Decision tree for the middle-aged group.

**Figure 6:**
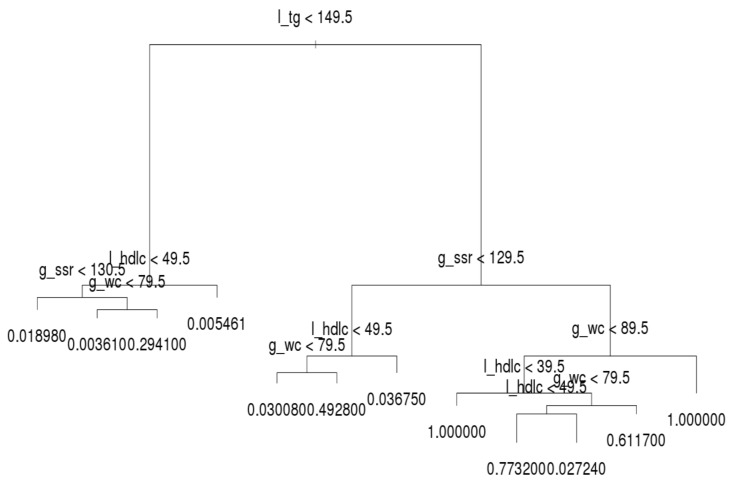
Decision tree for the older group.

The decision trees for the three age groups are presented in Figure 4, Figure 5 and Figure 6, which shows that l_tg was not always the leading factor at the top of the decision tree. Instead, the factor, g_wc, was more crucial in the younger group than other groups. Hence, younger persons are recommended to control their g_wc in order to prevent MetS.

Regarding the younger group, l_tg was the second most crucial factor. If a younger person has normal g_wc and l_tg, he or she only has a 0.16% likelihood of acquiring MetS. If a person’s g_wc and g_ssr are normal and l_tg is abnormal, the likelihood of acquiring MetS increases from approximately 4.26% to 11.16%. When the other factors on the left-hand side of the tree are abnormal, the individual in question meets the criteria for MetS. On the right-hand side tree in the younger group, l_tg is the second most crucial criterion. However, the third and fourth most crucial criteria differ under l_tg; l_hdlc is the third node over g_ssr.

For the middle-aged group, the structure of the decision tree was identical to that of the whole population (Figure 5). In addition, their MetS values were quite similar. The number of records and rate of MetS were both high in the middle-aged group. The behavior of the decision tree was dominated by the middle-aged group; this is why the tree structure of the middle-age group was identical to that of the whole population.

For the older generation, l_tg was the leading node in the decision tree (Figure 6), similar to the middle-aged group. However, the second to fourth factors were entirely different on the left- and right-hand sides of the trees. Researchers and practitioners are recommended to observe these trees in detail.

## 4. Discussion

Several studies on MetS have focused on tracking patients with diabetes or cardiovascular disease (CVD), and more recent studies on MetS have focused on healthy or sub-healthy people [19,20,21]. Kuk and Chris [19] demonstrated that MetS is a heterogeneous entity with age and sex variation in component clusters that may have important implications for interpreting the association between MetS and mortality risk. Hildrum et al. discovered that the prevalence of MetS increased strongly with age according to the ATP III and International Diabetes Federation [20]. Younger people with MetS in rural Victoria, Australia, may have warranted more aggressive preventive treatment for CVD than that suggested based on the summation of their individual risk factors [21].

Results of data collection and analysis regarding MetS from the MJPD have been published [8,22,23]. In Taiwan, where data from health examinations were utilized, a 12.5% 5-year cumulative incidence of MetS and increased occurrence of MetS in women with more limited education as they became older were observed [23]. Liao and Lin [8] tested and analyzed the potential health effects of various factors by using multiple logistic regression and a generalized estimating equation model. A 10% increase in MetS prevalence was observed over the 9-year period of the study. The mean Framingham CVD score for people with MetS was estimated to be approximately 1.4% (standard deviation (SD) = 1.5%) [8]. Huang et al. [22] demonstrated that MetS is a common disorder among Taiwanese adults and is similarly associated with an increase in all-cause and CVD mortality, similar to Western populations. Asians with MetS are at higher risk of death. Our study focused on assessing the MetS indicators in the ATP III and discovered the most crucial factors for preventing MetS. Therefore, our research goals differed to those of the aforementioned three studies. 

From the perspective of public health and preventive medicine, most experts or professionals consider that FG, WC, BP, and FPG are the most crucial risk factor indices for MetS [3,14]. Variation exists in how different combinations of MetS risk factors are associated with mortality. Kuk and Chris [19] applied the five criteria of the ATP III to analyze five major indicators of each combination of MetS indices. In older women, elevated glucose (high FPG) or low HDL-C as a MetS component was most strongly associated with mortality risk. Devers et al. (2016) reported that the prevalence of MetS (followed by the ATP III criteria) among people with normal WC was lower than expected [21]. 

However, TG was the most significant contributor to MetS in our analysis according to the decision tree classification results. A decision tree algorithm indicates the priority of the influence factors in a row. This finding was similar to that of a previous study, which suggested that TG—the root node—was the most informative factor for MetS identification in Thailand [6]. Lemieux et al. [24] noted that hypertriglyceridemic waist is a predictor of metabolic abnormalities. The preceding two studies support our analysis regarding the importance of leading branch indicators. Our study results for the healthy middle-aged and sub-healthy groups indicated that if the first two criteria (TG and WC) are excluded from the decision tree, the risk of MetS is lower. Therefore, high TG and WC are key factors for preventing MetS among middle-aged and elderly people; these factors are often overlooked by health management experts and require further verification. Because we employed a large dataset, our analysis results might be sufficient to comprehensively show the phenomenon of people with MetS.

After our discussions with a cardiologist and a physician specializing in metabolism, we estimated that the TG level varies greatly in in the daily diet of people. Mensink and Katan [25] published an article on a meta-analysis of 27 trials, and they reported that dietary fatty acids intake resulted in a more stable level of serum HDL than TG. Other studies have also indicated that medium-chain fatty acids or medium-chain TG levels are altered or affected by daily diet [26,27,28]. Asakura et al. [29] found that the total plasma cholesterol levels did not change in response to a liquid test meal with corn oil, but TG levels increased with a 100% corn oil diet.

TG values seem to be influenced by daily diet. However, because our dataset was provided by the Major Health Screening Center, the individuals therein were advised to fast for 6–8 h before undergoing an annual health examination. TG values drop during this 6–8 h period. A patient’s TG level is controllable before he or she receives an examination; however, some of the people analyzed in this study still had high TG levels. Therefore, TG is a strong prediction index for preventing MetS.

## 5. Conclusions

MetS is a growing public health concern in the United States and Taiwan because it increases the risks of CVD, type 2 diabetes mellitus, and stroke [9,10,11,12,13,30]. In addition, MetS is highly correlated with overweight status or obesity. When a person does not meet the criteria for MetS, he or she has a low likelihood of developing problems such as CVD and type 2 diabetes mellitus or becoming overweight. Although MetS is a pertinent research topic and has been studied extensively, the explicit importance of the factors defined by the ATP III are unknown. This study investigated the key factors determining MetS.

We applied a decision tree algorithm to identify the importance of the five criteria of the ATP III for identifying MetS symptoms, namely WC, high BP (g_ssr; including SBP and DBP), FG, TG, and HDL-C. The first notable finding was that not all criteria were equally crucial, and FG was not even used in the decision tree.

The second meaningful finding was that TG is the root factor for the whole population. If a person manages their TG level, they have a lower likelihood of developing MetS; the probability of MetS development is only 1.1% instead of 7% of the total average. A person with abnormal TG levels must control their WC first and then control their BP and HDL-C. This guideline can be followed for MetS prevention.

We examined the details of the behavior of the three age groups. The results revealed that the entire tree structure of the middle-aged group was equivalent to that of the whole population because middle-aged people accounted for the highest number of observations and highest number of MetS patients. However, we observed some differences between the younger and older age groups. For example, for a younger person, the root factor was WC and the second most explicit factor was TG. Regarding the tree structure of the older generation, although the root factor was TG, older people must manage their HDL-C or BP as a secondary explicit factor if their TG levels are normal or abnormal, respectively. Therefore, maintaining a low TG level may be a vital factor for MetS prevention in middle-aged and healthy older people. We hope that this finding will help in the prediction and management of patients with MetS; however, this conclusion requires support from further clinical studies or longitudinal cohort studies. The provision of comprehensive and integrated clinical management for patients with MetS or diabetes is vital.

In future research, we intend to target health examination groups with a long-term observation and analyze their TG levels. Compared with other indicators (FG, WC, BP, and FPG), an increase in TG will more likely increase the risk of MetS in secondary, tertiary, and quaternary health examination reports. In addition, we intend to analyze longitudinal cohort studies and the occupations of patients with MetS. Because the dataset in this study comprised more than 70 features, exploring the association between MetS and occupation to determine cost-effective treatment methods for MetS may be appealing.

## Figures and Tables

**Table 1:** Criteria for metabolic syndrome (MetS) defined by the modified Adult Treatment Panel III (ATP III).

Number	Criterion	Abnormal Condition
1	Waist circumference (WC)	Male ≥ 90 cm or Female ≥ 80 cm
2	High blood pressure (BP)	SBP ≥ 130 mmHg or DBP ≥ 85 mmHg
3	Fasting plasma glucose (FG)	FG ≥ 100 mg/dL
4	Triglyceride (TG)	TG ≥ 150 mg/dL
5	High density lipoprotein cholesterol (HDL-C)	Male < 40 mg/dL or Female < 50 mg/dL

**Table 2:** Number of MetS cases in the three age groups.

Group	Total	MetS Records	Average
Younger	87,289	4258	5%
Middle	10,0170	7901	8%
Older	13,628	1926	14%
Total	201,087	14,085	7%
